# Winnipeg a Good Example

**Published:** 1897-08

**Authors:** 


### EDITORIAL.

### WINNIPEG A GOOD EXAMPLE.

THROUGH the efforts of her earnest, well-informed veterinarians Winnipeg enjoys to-day the most complete safeguards of her milk-supply. From the source of production, the animals that furnish it, the seller or vendor in the street, and the shops where it is distributed, not an avenue of danger is left unguarded, and the milk-consumers of that city have more to be thankful for than any large city of North America. These regulations have been compiled in pamphlet form, and every city intending to better guard the interests of its people should study well the regulations of Winnipeg.

Manitoba in general seems to be a happy realm for veterinarians. Her people have learned of their value, and have been eager at all times to avail themselves of the best and broadest services the profession has had to give. Acting upon this plan they are better protected in their food-supply; they are freer from impostors and quacks than any other country, and no just estimate can be made of what they are saved from in material losses through efficient and intelligent veterinary services. All honor to the people of Manitoba, and all praise to her earnest veterinarians!

